# Design and Implementation of a Real-Time Visual Tracking System for UAVs Based on PSDK

**DOI:** 10.3390/s26072145

**Published:** 2026-03-31

**Authors:** Ranjun Yang, Ningbo Xie, Qinlin Li, Kefei Liao, Jie Lang, Kamarul Hawari Bin Ghazali

**Affiliations:** 1 School of Information and Communication, Guilin University of Electronic Technology, Guilin 541004, China; 24022304015@mails.guet.edu.cn (R.Y.); xieningbo@guet.edu.cn (N.X.); liqinlin@guet.edu.cn (Q.L.); langjie@guet.edu.cn (J.L.); 2 Joint International Research Laboratory of Spatio-Temporal Information and Intelligent Location Services, Guilin University of Electronic Technology, Guilin 541004, China; 3 Center for Advanced Industrial Technology, Universiti Malaysia Pahang Al Sultan Abdullah, Pekan 26600, Malaysia; kamarul@umpsa.edu.my

**Keywords:** unmanned aerial vehicle (UAV), visual tracking, edge computing, visual servo control, DJI

## Abstract

**Highlights:**

**What are the main findings?**

- We abandoned the ROS layer to eliminate its inherent communication overhead and utilized DJI PSDK to construct a middleware-free architecture, thereby reducing end-to-end latency by over 60% (~29 ms).
- We proposed a robust perception-control framework integrating the ADVS algorithm, multi-criteria fusion strategy, and FSM logic; field tests validated the efficacy of the dead-zone logic and achieved high-precision tracking (RMSE 0.028).

**What are the implications of the main findings?**

- The implementation confirms that achieving high-performance edge tracking does not rely solely on scaling visual model complexity, but it can also be effectively achieved through architectural latency minimization and robust control strategies.
- The study demonstrates a viable paradigm for autonomous UAVs where the synergy of the ADVS algorithm, multi-criteria fusion strategy, adaptive dead-zones, and FSM logic strictly enforces flight safety in dynamic environments.

**Abstract:**

This paper presents the design and implementation of a real-time visual tracking system for unmanned aerial vehicles (UAVs), based on the DJIPayload Software Development Kit (PSDK), addressing the challenge of balancing high precision with low latency on resource-constrained edge platforms. By utilizing DJI PSDK to abandon the Robot Operating System (ROS) layer and its associated serialization overhead, the proposed Middleware-Free Architecture reduces end-to-end latency by over 60% to approximately 30 ms. To address computational constraints, a Lightweight Asymmetric De-coupled Visual Servoing (ADVS) strategy is proposed. It adopts orthogonal kinematic de-coupling to bypass Jacobian matrix inversion and integrates a non-linear dead-zone mechanism with dynamics-aware gain scheduling to compensate for sensing anisotropy and gravitational nonlinearity. Simultaneously, a Geometry-Aware Fusion strategy is employed to reject visual outliers, while a Finite State Machine (FSM) strictly enforces temporal consistency. Field experiments in various scenarios verify the system’s stability and tracking capability. Specifically, the platform maintains a robust lock on targets at speeds up to 23 m/s across dynamic maneuvers. The successful implementation of this system confirms that high-performance edge tracking does not rely solely on the scaling of visual model complexity but can also be effectively achieved through the architectural minimization of latency combined with the optimization of theoretically grounded robust control strategies.

## 1. Introduction

With the rapid proliferation of Unmanned Aerial Vehicle (UAV) technology, vision-based autonomous tracking has become essential in urban security, search and rescue, and infrastructure inspection [1,2]. While the demand for robust, real-time autonomy is critical, existing solutions face significant challenges in balancing algorithmic sophistication with the strict Size, Weight, and Power (SWaP) constraints of embedded platforms [3].

Current research predominantly focuses on maximizing detection metrics through high-complexity models, often overlooking the latency constraints of real-time edge deployment. For instance, recent studies have introduced global agent-based transformers [4] and bi-level routing attention mechanisms [5] to enhance tracking precision and small target recall. Similarly, Vu et al. [6] combined slicing-aided hyper inference with YOLOv11 to improve small object detection. However, the multi-stage processing inherent in these methods introduces substantial latency, rendering them impractical for resource-constrained edge devices. Computational complexity analysis suggests that deploying such architectures on platforms like the Jetson Nano would likely result in single-digit frame rates [7]. However, as highlighted in recent research bridging theory to applications [6], relying solely on computer vision presents inherent limitations regarding environmental robustness and computational trade-offs. Pure vision-based approaches remain susceptible to environmental perturbations [6,8]. Factors such as motion blur and complex backgrounds [6], combined with high-frequency UAV vibration and rapid altitude fluctuations, degrade feature extraction, leading to diminished confidence. Without robust logic to manage these “hard negative” scenarios, tracking drift is inevitable. Although deep reinforcement learning has been explored to maintain target persistence [9], a significant body of research remains confined to idealized simulation environments. For instance, platforms like Aerial Gym [10] and OmniDrones [11] prioritize massive parallel training for high-dynamic tracking but operate purely within GPU-accelerated virtual environments. Similarly, platforms like Flightmare [12] and specialized extensions such as Agri-fly [13] provide high-fidelity visual rendering for complex semantic tracking tasks, yet they systematically bypass the stochastic aerodynamic disturbances and non-ideal sensor noise found in real-world flight. Deploying such computationally intensive frameworks on standalone edge units remains a formidable engineering challenge.

From a system architecture perspective, most UAV solutions rely on the Robot Operating System (ROS) middleware. While ROS offers modularity, its standard communication mechanisms impose significant overhead on high-bandwidth data transmission. Empirical benchmarks indicate that end-to-end latency in ROS-based systems frequently exceeds 100 ms due to message serialization and processing delays [14]. In high-speed tracking, this latency creates a critical “blind distance,” severely compromising control stability. Furthermore, traditional visual servoing often employs symmetric proportional control, neglecting the asymmetric vertical dynamics of multi-rotor UAVs—specifically, the high thrust required to overcome gravity during ascent versus the gravity-assisted nature of descent. Ignoring this physical reality results in sluggish ascent and overshoot during descent.

Beyond dynamic constraints, position-based control strategies—ubiquitous in waypoint navigation—exhibit fundamental vulnerabilities [15]. These methods rely heavily on global state estimation, which is prone to cumulative drift in GPS-denied or texture-poor environments [16]. A critical failure mode is “set-point latching”: if the visual tracker suffers temporary occlusion, the position controller often defaults to the last valid coordinate. Unlike velocity-based paradigms that inherently damp inertia, this behavior lacks an immediate interrupt mechanism, frequently precipitating “fly-away” incidents where the UAV accelerates toward an invalid target [17].

To mitigate these challenges, this paper proposes a real-time tracking system that bridges the gap between high-performance industrial platforms and onboard edge computing. Adopting an “Edge Computing + Deep Integration” methodology, we utilize YOLOv8 as the core detection module, selected for its optimal balance between inference speed and mean Average Precision (mAP) on the Jetson Orin NX platform. Distinct from traditional architectures, “Deep Integration” refers to a middleware-free communication mechanism achieved via the DJI Payload Software Development Kit (PSDK). This mechanism establishes a closed-loop perception-control architecture with millisecond-level response.

The main contributions of this paper are summarized as follows:

1. A Middleware-Free Low-Latency Architecture via PSDK: Diverging from traditional ROS-based architectures that rely on TCP/IP loopback communication and serialization, this paper implements a direct architecture using the DJI PSDK. By encapsulating control commands via direct API calls, the system eliminates intermediate forwarding layers. This design reduces the end-to-end communication latency to approximately 30 ms, demonstrating the feasibility of high-frequency control on edge platforms;
2. Lightweight Asymmetric Decoupled Visual Servoing (ADVS) Control Algorithm: To address the computational limits of embedded units, this paper proposes a lightweight ADVS algorithm as an alternative to complex Jacobian-based IBVS methods. Utilizing an Orthogonal Kinematic Decoupling strategy, the system designs asymmetric proportional coefficients combined with a three-axis decoupled control law. This approach bypasses the computational overhead of matrix inversion while ensuring a smooth and responsive tracking performance;
3. A Robust Perception-Control Strategy Based on Multi-Criteria Fusion: To overcome the inherent uncertainty and signal jitter of detection algorithms in unstructured environments, a hierarchical robustness strategy is established. First, a Geometry-Aware Multi-Criteria Fusion Logic—integrating geometric constraints with detection confidence scores—is designed to strictly reject visual outliers at the perception stage. Second, an adaptive dead-zone mechanism is proposed to suppress pixel-level control oscillations, alongside a Finite State Machine (FSM) to ensure deterministic state transitions and mission continuity during temporary target loss.

## 2. System Overview and Architecture Design

### 2.1. Hardware Architecture and Safety Redundancy

**Figure 1:**
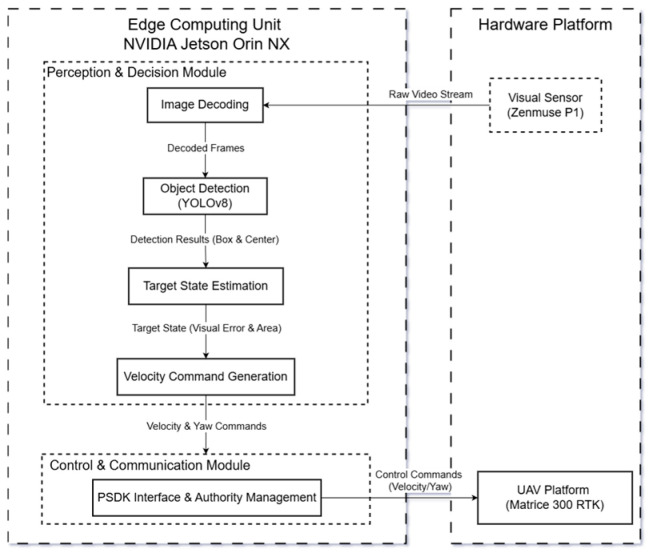
System framework diagram.

The architecture of the proposed autonomous tracking system is illustrated in Figure 1. The Zenmuse P1 captures and transmits raw video streams to the edge unit. The NVIDIA Jetson Orin NX employs YOLOv8-based monocular perception for target state estimation, extracting visual error and bounding box area. Resultant control commands are synthesized and relayed via PSDK to the UAV platform for execution.

The system adopts a decoupled architecture, assigning high-level perception tasks to the independent edge unit while reserving low-level attitude stabilization for the flight controller. The rationale for this separation lies in safety redundancy: in the event of vision algorithm failure or CPU/GPU saturation on the edge device, the flight controller maintains independent control authority, thereby acting as a fail-safe to prevent catastrophic flight failure.

### 2.2. Algorithm Selection and Perception Module

YOLOv8 is selected as the core perception algorithm for the Jetson platform following a rigorous comparative analysis of the recent literature. As summarized in Table 1, while Transformer-based models (e.g., RT-DETR) offer superior accuracy, they incur excessive memory overhead that compromises real-time edge performance [18]. Similarly, newer architectures (YOLOv10/v11) exhibit optimization gaps in TensorRT support or diminishing returns regarding small object recall [19,20,21]. In contrast, YOLOv8 outperforms the legacy YOLOv5 by leveraging the C2f module for enhanced feature aggregation [22]. Consequently, as the comparative data indicates, YOLOv8 is adopted for its optimal balance of inference stability, ecosystem maturity, and speed [7].

### 2.3. Middleware-Free Communication Mechanism

**Figure 2:**
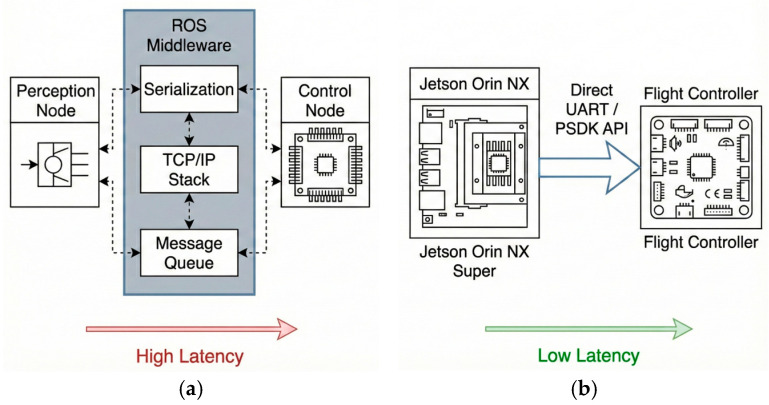
Comparison of communication architectures: (**a**) Traditional ROS-based framework showing middleware overhead. The dashed arrows represent the internal data flow and inter-process communication pathways between the functional nodes and the ROS middleware; (**b**) Proposed middleware-free “Deep Integration” via direct PSDK API.

In this study, “Deep Integration” is defined as a middleware-free, function-level call mechanism circumventing the standard operating system network stack. Unlike traditional ROS-based architectures necessitating TCP/IP loopback and data serialization, the proposed system operates directly at the driver interface level. Figure 2 illustrates the bottleneck elimination mechanism. Conventional setups are constrained by the buffering of the Linux kernel’s “ftdisio” driver. To optimize throughput, this driver enforces a default 16 ms latency timer [23], which, combined with ROS middleware serialization, results in cumulative latency often exceeding 60 ms [24].

The proposed architecture implies a “Deep Integration” strategy that bypasses the standard OS network stack. As detailed in Table 2, this approach eliminates the middleware layer by accessing the UART interface directly via the PSDK API. From a system perspective, this constitutes a structural optimization: the traditional ROS architecture suffers from a cumulative latency (>60 ms) stemming from Linux kernel’s “ftdi_sio” driver buffering (default 16 ms timer) [23] and the significant serialization overhead inherent to the ROS communication stack [24]. In contrast, according to standard transmission principles [25], the physical transport latency for raw binary data is theoretically less than 1 ms. By architecturally removing these serialization bottlenecks, the proposed system stabilizes the end-to-end latency at approximately 30 ms (experimentally measured, accounting for camera exposure, inference, and transmission). Consequently, this architectural optimization effectively reduces the total system latency compared to the theoretical baseline.

## 3. Methodology

**Figure 3:**
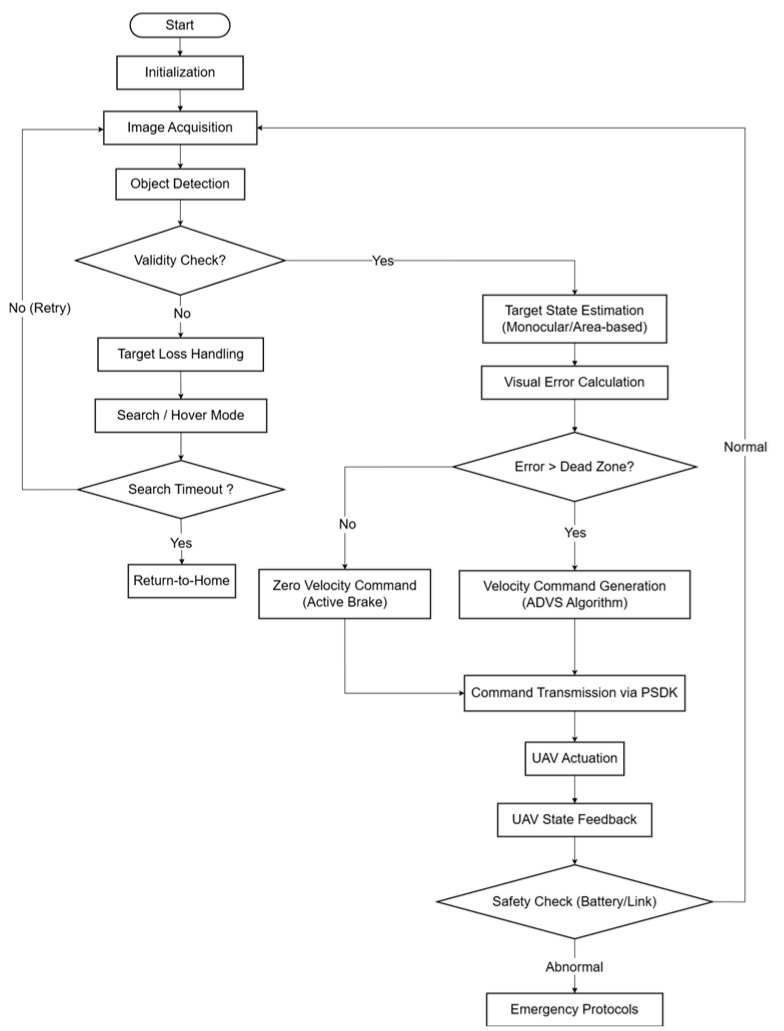
System flowchart.

The proposed system employs a closed-loop feedback control architecture designed for real-time operation on resource-constrained hardware. As illustrated in the system flowchart (Figure 3), the operational logic follows a sequential four-stage execution:

The workflow initiates with model initialization and image acquisition, followed by object detection and validity verification. Invalid detections activate the Finite State Machine (FSM), which executes search protocols or a timeout-driven Return-to-Home (RTH) failsafe. Valid inputs are processed for monocular state estimation and error computation. Subsequently, visual errors are evaluated against an adaptive dead-zone: errors within the threshold trigger an “Active Brake” command (v=0) to mitigate oscillation, while those exceeding it engage the ADVS algorithm for asymmetric velocity generation. Final control commands are encapsulated and transmitted via PSDK to the flight controller. Real-time status monitoring ensures continuous cycle iteration or triggers emergency intervention upon anomaly detection.

### 3.1. UAV Kinematic Modeling and Decoupling Strategy

#### 3.1.1. Kinematic Abstraction Based on Time-Scale Separation

The full dynamics of a quadrotor are governed by the coupled 6 DoF Newton–Euler equations [26]:

(1) $$\left\{\begin{array}{ll} \dot{p}=v & \\ m\dot{v}=R_{B}^{W}f_{T}-mg-Dv & \\ J\dot{\omega }=\tau -\omega \times \left(J\omega \right) & \end{array}\right.$$

where p, v, ω denote the position, linear velocity, and angular velocity; m, J represent the mass and inertia matrix; $R_{\left|B\right|}^{\left|W\right|}$ is the rotation matrix; and $f_{\left|T\right|}$, τ, g, D correspond to the thrust, torque, gravity, and drag terms, respectively. To simplify the control architecture for real-time visual servoing, the Time-Scale Separation Principle is applied. Given that the bandwidth of the inner attitude loop $\left(BW_{\mathrm{att}}\right)$ provided by the industrial flight controller significantly exceeds that of the outer guidance loop $\left(BW_{\mathrm{pos}}\right)$ [27]:

(2) $$BW_{\mathrm{att}}\;\gg BW_{\mathrm{pos}}\Rightarrow \tau_{\mathrm{att}}\;\approx 0$$

Under this condition, the high-order non-linear dynamics can be abstracted into a lower-order kinematic model. The complex relationship between thrust/torque and velocity is reduced to a first-order linear response. While Bauer et al. [28] identified high-fidelity dynamics for DJI platforms, for the specific purpose of visual guidance, the closed-loop behavior is effectively approximated as a first-order lag process:

(3) $${\dot{v}}_{B}\approx \Lambda \;\left(u_{\mathrm{cmd}}-{\;v}_{B}\right)$$

where $v_{B}$ denotes the body-frame velocity state, $u_{\mathrm{cmd}}$ is the virtual control input, and Λ is the diagonal gain matrix. This abstraction allows the UAV to be treated as a velocity-controlled agent, obviating the need to solve full dynamic equations onboard.

#### 3.1.2. Lightweight Asymmetric Decoupled Visual Servoing (ADVS) Control Algorithm

**Figure 4:**
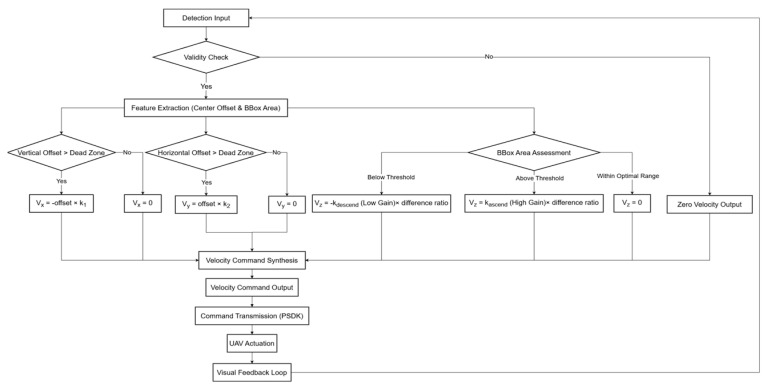
Flowchart of the ADVS Algorithm.

Traditional position-based controllers minimize errors in 3D Cartesian space but are intrinsically limited by cascade-induced latency and susceptibility to state estimation drift in unstructured environments [16]. Additionally, the precise derivation of the image Jacobian matrix demands continuous depth estimation; on resource-constrained embedded platforms, the iterative inversion of this matrix imposes computational latency that can destabilize high-frequency loops. To circumvent these bottlenecks, and building upon the kinematic abstraction in Section 3.1.1 which models the UAV as a velocity-controlled system, the proposed Lightweight ADVS algorithm directly maps visual errors to velocity commands. Implementing an Orthogonal Kinematic Decoupling architecture (as illustrated in Figure 4) the system decomposes the coupled 3D tracking task into three independent scalar channels ($u\to v_{y},v\to v_{x},S\to v_{z}$). This design eliminates the overhead of Jacobian inversion, prioritizing deterministic, low-latency execution. Crucially, this strategy decouples tracking performance from global positioning accuracy, enabling the Finite State Machine (FSM) to execute an instantaneous “Active Brake” (v=0) upon target loss—a critical feature for ensuring flight safety in high-dynamic environments [29].

**Figure 5:**
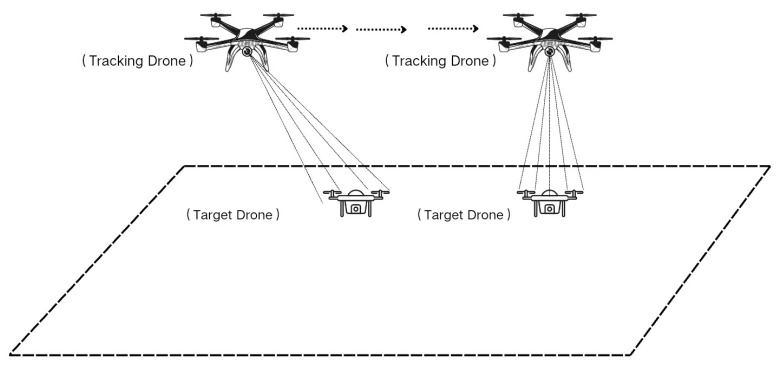
Schematic diagram of the tracking scenarios.

To clarify the spatial relationships, Figure 5 illustrates the nadir-viewing (downward-facing) configuration. The image horizontal axis (u) governs lateral movement ($v_{y}$), the image vertical axis (v) governs longitudinal movement ($v_{x}$), and the target scale (S) governs vertical altitude ($v_{z}$).

Upon identifying a target, the system extracts the bounding box coordinates. Let $\left(x_{0},y_{0}\right)$ denote the top-left pixel coordinates, and $\left(w,h\right)$ represent the width and height. Consequently, the bounding box center $\left(u,v\right)$ is calculated as follows:

(4) $$\left(u,v\right)=\left(x_{0}+\frac{w}{2},y_{0}+\frac{h}{2}\right)$$

The projection from the 3D camera frame to the 2D image plane is governed by the intrinsic matrix K:

(5) $$K=\left[\begin{array}{ccc} f_{x} & 0 & c_{x}\\ 0 & f_{y} & c_{y}\\ 0 & 0 & 1 \end{array}\right]$$

Here, $f_{x}$ and $f_{y}$ represent the focal lengths in the pixel, and ($c_{x}$, $c_{y}$) denotes the coordinates of the principal point. In tracking scenarios where active ranging sensors or stereo cameras are unavailable, monocular vision is adopted. To resolve the inherent scale ambiguity, a geometric prior is introduced. Specifically, within the effective operational range ($Z_{c}\in \left[2,50\right]m$), the depth is recovered using the Principle of Similar Triangles by constraining the target’s physical width $W_{phy}$ to a known average value (field tests indicate that dimensional deviations among typical models do not significantly compromise tracking stability). Thus, the depth is estimated as

(6) $$Z_{c}=\frac{f_{x}\cdot W_{phy}}{w}$$

where w is the bounding box pixel width, and $f_{x}$ is the horizontal focal length derived from K to match the physical width $W_{phy}$. This geometric relationship ($Z_{c}\propto w^{-1}$) provides the theoretical basis for using the bounding box area (S=w⋅h) as a proxy for altitude control in the subsequent derivations.

In practical engineering scenarios, relying solely on raw pixel coordinates or noisy depth estimates can lead to control instability. To eliminate the influence of varying image resolutions and facilitate cross-platform applicability, we employ a normalization strategy based on image semi-dimensions. Let $W_{img}$ and $H_{img}$ denote the image width and height. The normalized visual offsets $\delta_{u},\delta_{v}\in \left[-1,1\right]$ are defined as

(7) $$\delta_{u}=\frac{u-c_{x}}{W_{img}/2},\;\delta_{v}=\frac{v-c_{y}}{H_{img}/2}$$

1. Planar Control ($v_{x}$, $v_{y}$) with Non-linear Noise Suppression;

To mitigate control jitter induced by pixel-level sensor noise, a non-linear dead-zone activation function is integrated into the control law. Unlike standard proportional control, this non-linearity acts as a signal gate, mathematically truncating the noise floor to enforce strict zero-velocity stability during target hovering. The $v_{x}$ and $v_{y}$ velocities are governed by

(8) $$v_{x}=\left\{\begin{array}{ll} -k_{1}\cdot \delta_{v}, & \left|\delta_{v}\right|>\delta_{\mathrm{th}}\\ 0, & Otherwise \end{array}\right.$$

(9) $$v_{y}=\left\{\begin{array}{ll} k_{2}\cdot \delta_{u}, & \;\;\;\;\left|\delta_{u}\right|>\delta_{\mathrm{th}}\\ 0, & \;\;\;\;Otherwise \end{array}\right.$$

where the dead-zone threshold is set to $\delta_{\mathrm{th}}\;=\;0.03$. This value was initially derived from the statistical analysis of the sensor noise floor (σ ≈ 0.008) following the standard 3σ principle (3σ ≈ 0.024) [30]. Through extensive field testing, the threshold was empirically refined to 0.03 to provide a conservative safety buffer, effectively filtering out measurement noise without compromising tracking sensitivity.

Furthermore, an axis-specific gain tuning strategy is adopted. This asymmetry is calibrated based on the sensor’s physical aspect ratio (typically 16:9)-reflecting the inherent disparity between the wider horizontal and narrower vertical fields of view [31]-the specific coefficients ($k_{2}\;=\;2.5$, $k_{1}\;=\;2.0$) were rigorously quantified through extensive flight testing. This design assigns a higher lateral gain ($k_{2}$) to effectively leverages the sensor’s wider horizontal Field of View (FOV) for agile tracking, whereas a conservative longitudinal gain ($k_{1}$) is selected to prevent the target from exiting the narrow vertical FOV during rapid braking maneuvers.

2. Asymmetric Altitude Control ($v_{z}$) with Dynamics Linearization;

Utilizing the inverse-square relationship derived above ($S\;\propto \;Z_{c}^{-2}$), the bounding box area is used as the feedback variable. An Asymmetric Gain Scheduling Strategy is proposed to address non-linear vertical dynamics [31]:

(10) $$v_{z}=\left\{\begin{array}{ll} -k_{\mathrm{descend}}\cdot \frac{S_{\min}-S}{S_{\min}}, & S<S_{\min}\;(\mathrm{Target}\;\mathrm{Distant})\\ k_{\mathrm{ascend}}\cdot \frac{S-S_{\max}}{S_{\max}}, & S>S_{\max}\;(\mathrm{Target}\;\mathrm{Close})\\ 0, & \mathrm{Otherwise} \end{array}\right.$$

Here, $k_{\mathrm{ascend}}$ and $\;k_{\mathrm{descend}}$ denote the ascending and descending gains, respectively. The variable S represents the real-time area of the target bounding box (in pixels), while $S_{\min}\;$ and $S_{\max}$ are the dynamic stability thresholds. To effectively counter the non-linear depth estimation and geometric coupling noise induced by target rotation [32], a relaxed dead-band strategy is adopted. As established in visual servoing theory [33], area-based metrics are inherently susceptible to geometric projection fluctuations (e.g., target yaw), resulting in a significantly lower signal-to-noise ratio compared to centroid features. To decouple this measurement noise from valid control signals, a relaxed tolerance margin is requisite. Consequently, the depth dead-zone was empirically optimized to $\delta_{d}^{\left(z\right)}\;=\;0.1$. This configuration effectively mitigates the resultant control oscillation, thereby ensuring stable station-keeping [34,35]. The stability thresholds are defined as

(11) $$S_{\min}=S_{\mathrm{ref}}\cdot \left(1-\delta_{d}^{\left(z\right)}\right)$$

(12) $$S_{\max}=S_{\mathrm{ref}}\cdot \left(1\;+\;\delta_{d}^{\left(z\right)}\right)$$

where $S_{\mathrm{ref}}$ is the reference target area configured to ensure the optimal tracking distance. Theoretical analysis indicates that ascending requires overcoming gravity (high thrust), while descending is gravity-assisted [36]. While theory dictates the necessity of linearization, the specific coefficients are quantified through extensive flight testing. Consequently, acting as a dynamics-aware linearization strategy, this paper assigns a higher gain $k_{\mathrm{ascend}}=1.5$ to compensate for the gravitational counter-force, and a lower gain $k_{\mathrm{descend}}=1.0$ to dampen the gravity-assisted inertia. This effectively linearizes the vertical control response in the closed-loop system.

The control outputs $\left(v_{x},v_{y},v_{z}\right)$ derived above represent dimensionless normalized commands rather than absolute physical velocities. To interface with the low-level flight controller, these normalized commands are mapped to actual physical execution velocities, denoted as $V_{\mathrm{physical},i}$ (m/s) through an actuator saturation constraint:

(13) $$V_{\mathrm{physical},i}=\mathrm{sat}\left(v_{i}\right)\cdot V_{\max,i},\;i\in \{x,y,z\}$$

where $V_{\max,i}$ denotes the maximum physical velocity limit of the UAV along the i-th axis. The saturation function is mathematically defined a $\mathrm{sat}\left(v_{i}\right)=\mathrm{sgn}\left(v_{i}\right)min\left(\left|v_{i}\right|,1.0\right)$. This mechanism ensures that if the calculated command exceeds the normalized limit ($\left|v_{i}\right|>1.0$), it is strictly bounded within the $\left[-1.0,1.0\right]$ interval, thereby commanding the UAV to track at its full-scale velocity limit ($V_{\max,i}$). Furthermore, this decoupled architecture—separating normalized control logic from platform-specific physical kinematics—guarantees the cross-platform adaptability of the proposed system.

### 3.2. Geometry-Aware Robust Perception Strategy Based on Multi-Criteria Fusion

Deep learning-based detection inherently introduces uncertainty in unstructured environments, frequently manifesting as High-Confidence False Positives (HCFPs) due to background clutter or visual aliasing [37,38]. To enforce deterministic physical consistency and mitigate probabilistic ambiguity, a Geometry-Aware Multi-Criteria Fusion Strategy is proposed.

Let $D_{k}\;=\;\{b_{1},b_{2},\ldots ,b_{N}\}$ denote the set of candidate detections at frame k. Each candidate $b_{i}$ is characterized by a measurement vector:

(14) $$b_{i}={\left[u_{i},v_{i},w_{i},h_{i},c_{i}\right]}^{T},$$

where $\left(u_{i},v_{i}\right)$ represents the center coordinates, $w_{i},\;h_{i}$ denote the width and height, and $c_{i}\;\in \;\left[0,\;1\right]$ is the confidence score. We define a Boolean Validity Indicator Function $\Phi :\;R^{5}\to \;\{0,\;1\}$ to filter outliers. The valid target set $D_{\mathrm{valid}}$ is logically derived as

(15) $$D_{\mathrm{valid}}=\{b_{i}\in D_{k}|\Phi \left(b_{i}\right)=1\}$$

The fusion function $\Phi \left(b_{i}\right)$ is constructed as the logical conjunction of three distinct physical constraints:

(16) $$\Phi \left(b_{i}\right)=I_{conf}\left(c_{i}\right)\wedge I_{scale}\left(w_{i},h_{i}\right)\wedge I_{shape}\left(w_{i},h_{i}\right)$$

1. Probabilistic Reliability Constraint ($I_{conf});$

To mitigate low-confidence noise while preserving recall, the probabilistic constraint is defined as a thresholding operation:

(17) $$I_{conf}\left(c_{i}\right)=\left\{\begin{array}{ll} 1, & c_{i}\geq c_{\mathrm{th}}\\ 0, & \mathrm{otherwise} \end{array}\right.$$

where $c_{th}=0.7$ is empirically determined based on the precision-recall curve derived from the validation dataset to optimize the trade-off between detection sensitivity and noise rejection.

2. Inverse-Square Geometric Scale Constraint $\left(I_{scale}\right)$;

Based on the pinhole projection model, the bounding box area $S_{i}=w_{i}\cdot h_{i}$ follows an inverse-square law relative to the target depth $Z_{c}$, given the target’s physical dimensions $\left(W_{phy},{\;H}_{phy}\right)$ [39]:

(18) $$S_{i}\approx \frac{\left(f^{2}\cdot W_{phy}\cdot H_{phy}\right)}{Z_{c}^{2}}\propto Z_{c}^{-2}$$

where ${\;S}_{i}$ is the bounding box area; $w_{i}$ and $h_{i}$ denote the width and height of the i-th candidate bounding box (in pixels); f denotes the camera focal length (approximated as $f{\approx f}_{x}\approx f_{y}$); $Z_{c}$ is the estimated target depth. Consequently, the feasible scale range is constrained by the operational safety range $\left[Z_{min},Z_{max}\right].$ The scale indicator is defined as

(19) $$I_{scale}\left(S_{i}\right)=I\left(S_{min}\leq w_{i}\cdot h_{i}\leq S_{max}\right)$$

This constraint effectively rejects distant background clutter $\left(S_{i}<S_{min}\right)$ and immediate collision risks $\left(S_{i}>S_{max}\right)$ [40].

3. Morphological Shape Constraint ${(I}_{shape}$);

Recognizing the rigid body characteristics of the quadrotor, its projected aspect ratio $r_{i}$ remains bounded despite perspective distortion during banking maneuvers [41]. The morphological constraint is formulated as

(20) $$I_{shape}\left(r_{i}\right)=I\left(r_{min}\leq \frac{w_{i}}{h_{i}}\leq r_{max}\right)$$

where $r_{i}$=$\;\frac{w_{i}}{h_{i}}$ denotes the aspect ratio of the candidate bounding box, and $\left[r_{min},r_{max}\right]$ represents the allowable aspect ratio interval derived from the UAV’s physical dimensions. This strictly distinguishes the target from morphologically distinct distractors.

### 3.3. Deterministic Finite State Machine (FSM) with Integral Hysteresis

While the perception layer filters spatial outliers, the temporal interaction between probabilistic detection sequences and deterministic flight control constitutes a Hybrid Dynamical System. As noted in robust visual servoing studies [42], direct coupling of these domains often induces “chattering” due to intermittent visibility or sensor noise. To address this instability, a deterministic Finite State Machine (FSM) incorporating a hysteresis-based switching logic is designed [43].

#### 3.3.1. Hysteresis-Based Transition Mechanism

To mitigate high-frequency oscillations during state switching, a Time-Integral Hysteresis Mechanism is implemented. Similar to the “Average Dwell Time” constraint, a transition from state $S_{i}$ to $S_{j}$ is validated only if the triggering condition $P_{s_{j}}$ persists for a duration $T_{\mathrm{hyst}}$. The transition validity $\tau_{\mathrm{trans}}$ is formally defined as

(21) $$\tau_{trans}\left(S_{i}\to S_{j}\right)=\int_{t_{now}-T_{hyst}}^{t_{now}}I\left[P_{s_{j}}\left(\tau \right)\right]\;d\tau \geq T_{hyst}$$

Here, $I\left[\cdot \right]$ denotes the indicator function, which takes the value 1 when the condition is true and 0 otherwise. This integral constraint functions as a temporal low-pass filter, rejecting transient false negatives. Additionally, a dynamic dead-zone logic is introduced to prevent actuator wear.

#### 3.3.2. Operational State Definitions and Parameter Configuration

**Figure 6:**
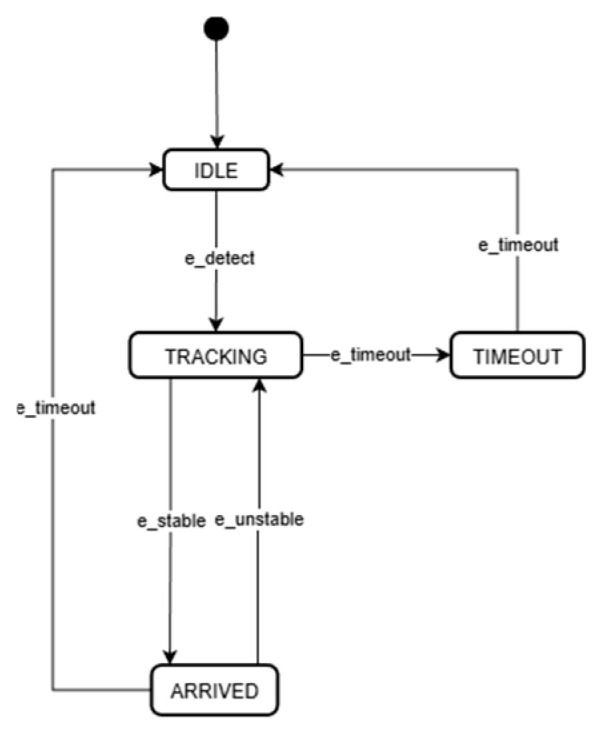
State transition diagram, The black dot represents the system’s starting point.

As illustrated in Figure 6, the mission logic is decomposed into four distinct phases.

1. State $S_{IDLE}$;

Handles initialization and “hard negative” scenarios (e.g., temporary loss). To ensure mission continuity, an Active Yaw-Spin Search Strategy is executed, setting velocity commands to $v_{IDLE}={\left[0,0,0,0.2\right]}^{T}$. to expand the Field of View (FOV).

2. State $S_{TRACKING}$;

Activated when the target is confirmed $\left(c_{i\;}\geq {\;c}_{\mathrm{th}}\right)$ and persistent. The ADVS control law (Section 3.1.2) is engaged with a control loop frequency synchronized at 25 Hz to align with the vision pipeline throughput.

3. State $S_{ARRIVED}$;

Signifies that the tracking error has converged within the dead-zone ($\delta_{\mathrm{th}}).$ The dead-zone threshold (0.03) acts as a “Zero-Order Hold” to eliminate steady-state chattering.

4. State $S_{\mathrm{TIMEOUT}}$;

Triggered if the target loss exceeds $T_{\mathrm{timeout}}$. This buffer allows for re-detection during complex maneuvers before resetting the system to prevent autonomous fly-aways.

Key control parameters, detailed in Table 3, are optimized through no less than 2.5 h of experimental flight time to balance system responsiveness with edge computing resource constraints.

## 4. System Implementation and Experimental Analysis

### 4.1. Hardware Integration and Latency Validation

**Figure 7:**
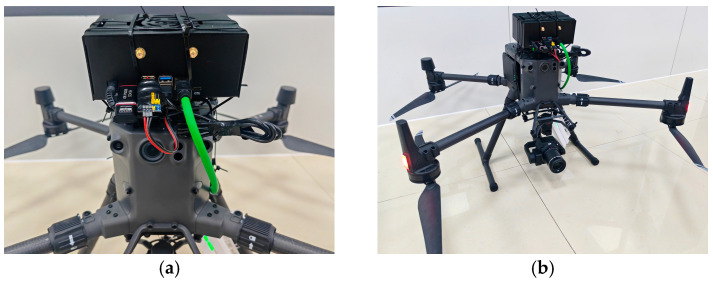
Hardware platform configuration: (**a**) Hardware interconnection diagram; (**b**) Overall system architecture.

The experimental platform utilizes the DJI Matrice 300 RTK industrial UAV. Onboard edge computing is performed by the NVIDIA Jetson Orin NX (Super) module, while visual perception is captured via the DJI Zenmuse P1 camera. The E-Port Developer Kit serves as the central communication bridge. Figure 7 illustrates the hardware topology and physical integration; the interface between the computing unit and the flight controller is established via a dedicated coaxial cable through the E-Port.

To quantify the real-time determinism of the proposed middleware-free architecture, we measured the end-to-end latency ($T_{total}$), defined as the time interval from raw image acquisition to PSDK API execution.

**Figure 8:**
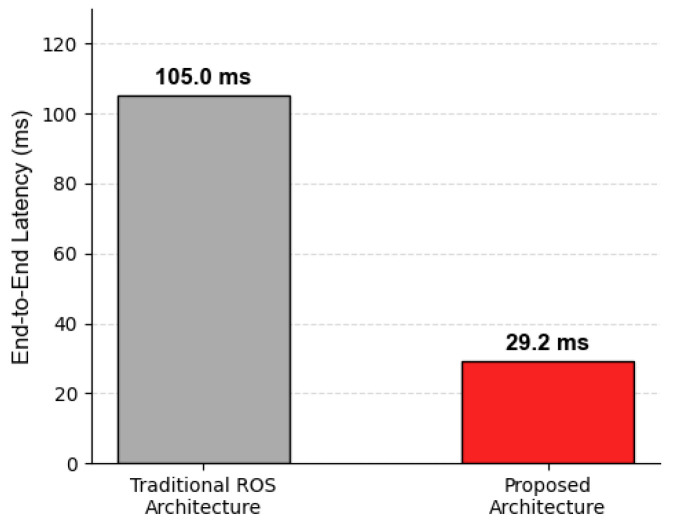
End-to-end latency comparison.

Figure 8 presents the end-to-end latency comparison. Consistent with the theoretical baseline (>60 ms) established in Table 2, the existing literature reports that standard ROS architectures typically incur latencies in the 50–100 ms range [44,45]. However, in practical scenarios involving high-bandwidth visual data serialization, these cumulative delays frequently escalate to approximately 105 ms due to chain-blocking effects. In contrast, the proposed PSDK-based integration stabilizes at approximately 29 ms. This represents a significant reduction of over 60% compared to the standard ROS framework, confirming that the middleware-free design effectively eliminates the stochastic overheads inherent to the operating system’s communication stack.

**Figure 9:**
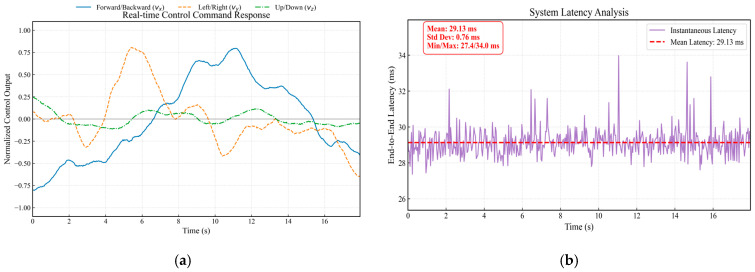
System response under dynamic loading: (**a**) Normalized control command input; (**b**) End-to-end latency stability.

Figure 9 further validates system stability under high computational loads. Figure 9a illustrates the control response during a complex tracking sequence involving significant negative pitch commands for retreating (t<4 s) followed by coupled lateral maneuvers (t>4 s). Despite this dynamic computational load, Figure 9b confirms that the end-to-end latency $\left(T_{total}\right)$ remains highly stable, with a mean of 29.13 ms and a standard deviation (σ) of only 0.76 ms. This minimal jitter confirms that the architecture effectively isolates computational dynamics from communication latency, satisfying the strict real-time requirements for high-speed tracking [46].

### 4.2. Simulation Environment and Preliminary Verification

**Figure 10:**
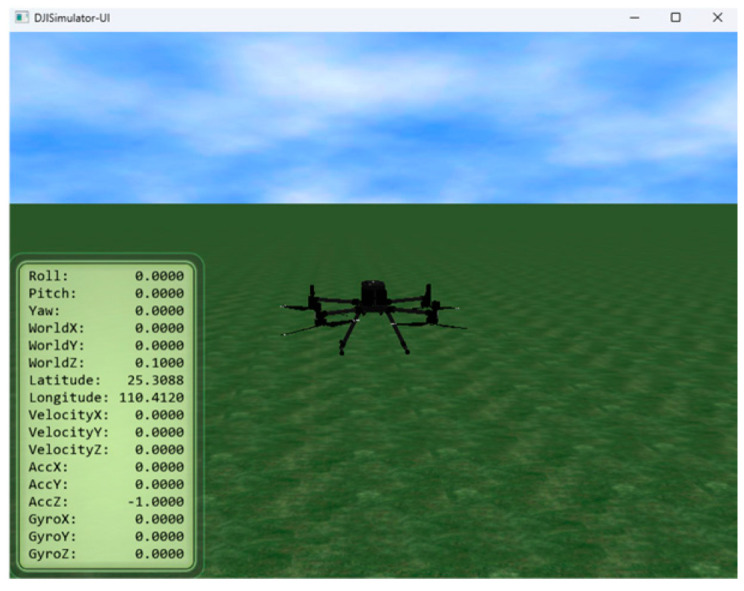
Interface of the DJI Assistant 2 simulator.

Prior to field deployment, simulation was performed using DJI Assistant 2. A stable communication link was confirmed between the Jetson Orin NX and the virtual Matrice 300 RTK. As illustrated in Figure 10, the system successfully received and parsed data transmitted by the flight controller. Critical flight parameters were correctly visualized in the interface, verifying the PSDK communication integrity.

**Figure 11:**
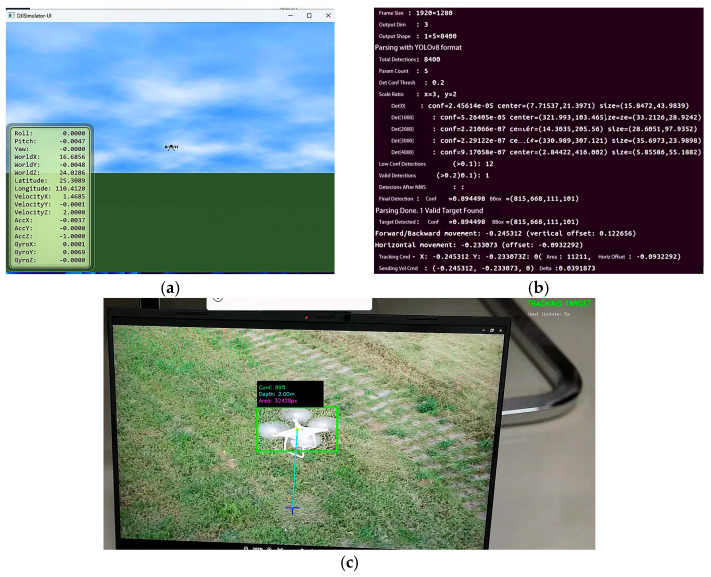
Simulation test results: (**a**) Flight view in the simulator; (**b**) Real-time data log on the edge computing platform; (**c**) Visual tracking and recognition interface.

To validate the perception–actuation loop, the system was tested under simulation tracking scenarios. As shown in Figure 11, quantitative analysis of the simulation data demonstrates that the system achieves robust target recognition, with detection confidence levels consistently exceeding 80%, ensuring the reliability of the control loop feedback.

### 4.3. Field Experiment Setup and Protocols

#### 4.3.1. Field Environment and Dataset

**Figure 12:**
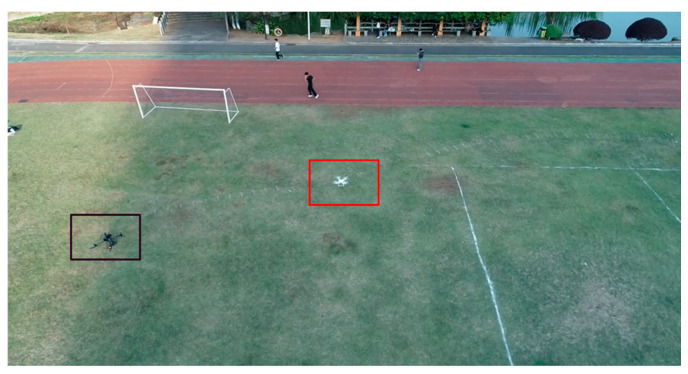
Panoramic view of the field test site. The black box on the left indicates the tracking platform, while the red box on the right highlights the target (DJI Phantom 4).

**Figure 13:**
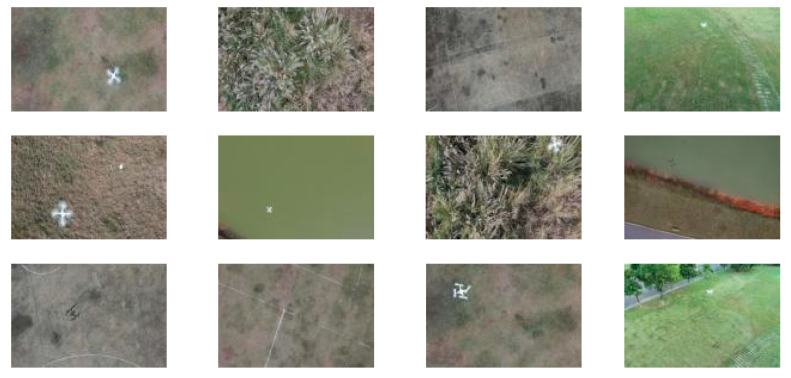
Representative samples from the constructed dataset covering various scenarios.

Field experiments were conducted in a spacious, unobstructed grassland environment to ensure favorable airspace conditions (Figure 12). To strictly adhere to safety protocols, a sparsely populated open area was selected to minimize potential risks. Additionally, to support the visual perception module, a specialized dataset comprising 10,000 high-resolution images was constructed. As illustrated in Figure 13, these samples cover diverse scenarios, varying distances, and complex backgrounds, ensuring the detection model’s generalization in the field.

#### 4.3.2. Definition of Tracking Scenarios and Challenges

To validate system robustness under real-world heterogeneity, the test campaign was designed to cover two critical dimensions: Target Heterogeneity and Environmental Complexity. As detailed in Table 4, the selection of test targets—DJI Mini 2 (Micro), Phantom 4 (Standard), and Inspire 2 (Medium)—establishes a physical gradient in size, weight, and speed, specifically designated to evaluate the system’s comprehensive robustness against physical variations.

Specifically, the Micro target (Mini 2), characterized by low inertia, is utilized to test detection sensitivity and the combined efficacy of the dead-zone logic and stability against wind-induced jitter. Conversely, the Medium target (Inspire 2) imposes boundary constraints: its larger dimensions occupy a significant portion of the image, challenging the system’s ability to prevent the target from exiting the Field of View (FOV) during close-range maneuvering. Furthermore, its high flight speed validates the system’s capability to minimize tracking lag (response delay) during rapid pursuit.

Although the comprehensive test campaign covered the aforementioned heterogeneous targets and extreme environmental conditions, to clearly elucidate the core control dynamics and algorithmic efficacy of the proposed system, the detailed waveform analyses presented in Section 4.4 are primarily derived from the DJI Phantom 4 under typical operational conditions (natural daylight with mild cloud cover). As shown in Figure 12, the bounding box on the left identifies the tracking platform, and the bounding box on the right marks the target (DJI Phantom 4).

#### 4.3.3. Experimental Design

The comprehensive field test campaign was executed through 20 independent flight sorties, accumulating approximately 4.5 h of effective closed-loop tracking time. Considering the operational constraints and battery endurance, this duration provides a substantial dataset covering continuous stable tracking phases. To verify system performance, four specific experimental scenarios were designed. Table 5 outlines the execution content for each experiment.

#### 4.3.4. Target Search and Acquisition Phase

**Figure 14:**
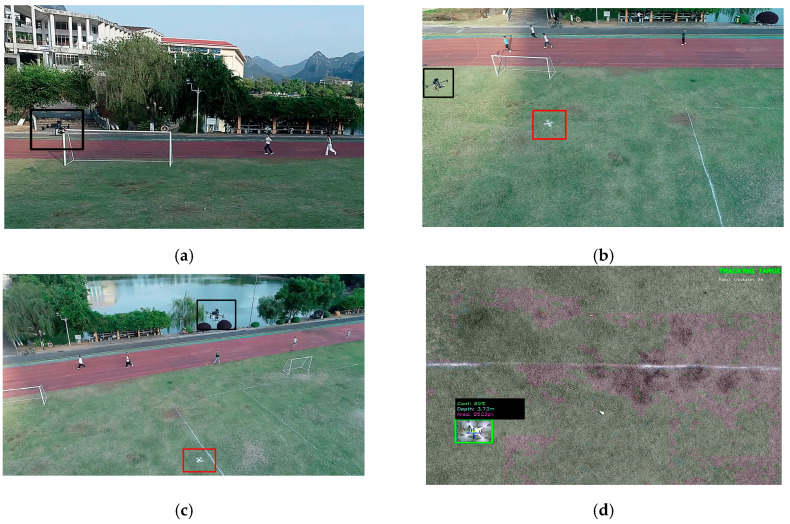
Sequence of system takeoff and target search: (**a**) System takeoff; (**b**) Executing search pattern; (**c**) Target identified (**d**) Real-time recognition status interface. In (**a**–**c**), the black bounding box indicates the tracking platform, while the red bounding box highlights the target (DJI Phantom 4).

Figure 14 verifies the autonomous transition from takeoff to active tracking in a real-world environment. As highlighted in Figure 14d, the ‘TRACKING TARGET’ status and real-time telemetry (confidence, distance, and area) confirm successful lock-on, validating the stability and effectiveness of the closed-loop architecture.

### 4.4. Dynamic Tracking Performance Analysis

#### 4.4.1. Planar Tracking Response Analysis

**Figure 15:**
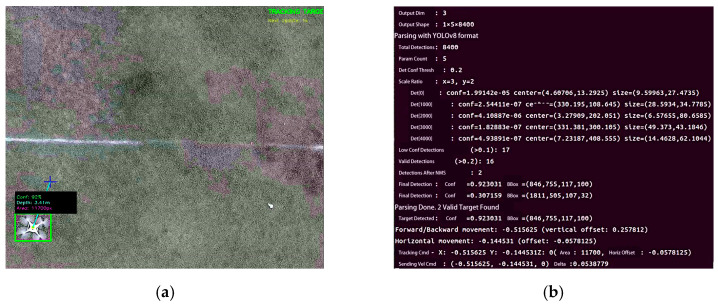
Backward tracking performance: (**a**) Visual recognition interface; (**b**) Edge computing platform terminal output.

In the planar tracking task, the primary objective is to maintain the target centered to minimize loss risks. Figure 15 illustrates the system’s response during high-dynamic maneuvers, where real-time compensatory velocity vectors are generated to rapidly recenter the accelerating target. The detection module consistently maintains confidence scores exceeding 92% while filtering noise candidates (<0.7), effectively confirming that the control law successfully maps visual offsets to kinematic commands.

To comprehensively evaluate the tracking precision in the 2D image plane, the Euclidean Norm $\left(L_{2}\right)$ of the normalized visual error vector is adopted as the performance metric:

(22) $$∥e\left(t\right)∥=\sqrt{e_{x}{\left(t\right)}^{2}+e_{y}{\left(t\right)}^{2}}$$

where $e_{x}$ and $e_{y}$ represent the horizontal and vertical normalized deviations. The Settling Time $\left(T_{s}\right)$ is quantitatively defined as the time required for the error magnitude to fall and stay below 10% of the initial peak error [47].

**Figure 16:**
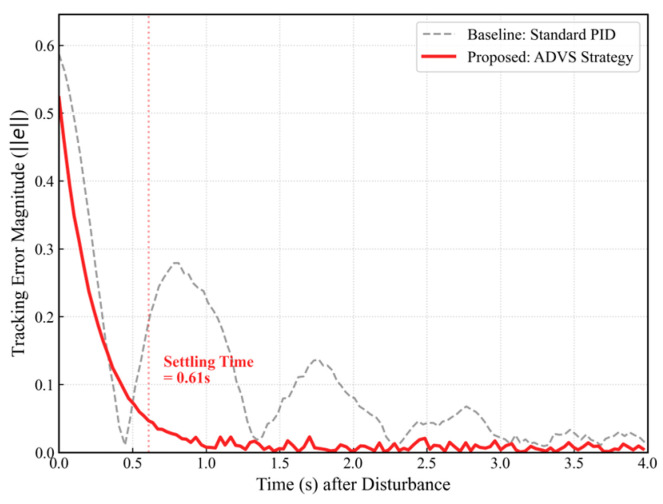
Convergence comparison. The vertical red dotted line indicates the settling time, defined as the moment when the tracking error first drops below 10% of its initial peak value.

Following the step-response protocol defined in Exp. I (Table 5), the system was subjected to a sudden large normalized visual error of approximately 0.5. The convergence trajectory of the proposed ADVS Strategy was compared against a Standard PID Baseline derived from real flight data. The error convergence comparison is illustrated in Figure 16, where the time axis is aligned such that (*t =* 0) corresponds to the moment of maximum disturbance.

As shown in Figure 16, the Standard PID baseline (gray dashed line) exhibits a relatively slow response accompanied by noticeable oscillations, resulting in a prolonged settling time exceeding 2.0 s. In contrast, the proposed ADVS strategy (red solid line) demonstrates a significant improvement in dynamic performance, achieving a rapid, monotonic descent in tracking error that effectively suppresses the oscillations observed in the baseline. The system reaches a stable state with a settling time of 0.61 s. This reduction in convergence time of over 60% verifies that the strategy successfully balances fast convergence with steady-state stability.

#### 4.4.2. Steady-State Error Distribution Analysis

To strictly evaluate the steady-state tracking precision, this study analyzes the systematic visual error recorded by the onboard mission computer. The error metrics are defined as dimensionless values derived from normalized image coordinates within the range $\left[-1,1\right]$, distinct from raw pixel counts or metric distances. The control objective is to minimize the normalized deviation between the target’s geometric center and the image principal point. This normalization strategy renders the control performance invariant to specific camera resolutions. Let $e_{x,i}$ and $e_{y,i}$ denote the normalized horizontal and vertical deviations for the i-th frame, respectively.

Given the dead-zone mechanism, the recorded error primarily reflects the dynamic residual—defined as the active oscillation occurring beyond the dead-zone threshold ($\delta_{th})$. Consequently, the tracking precision is quantified using the Root Mean Square Error (RMSE) of this residual component:

(23) $$RMSE=\sqrt{\frac{1}{N}\sum_{i=1}^{N}\left(e_{x,i}^{2}+e_{y,i}^{2}\right)}$$

Additionally, a Tracking Convergence Rate (η) is introduced to measure the percentage of frames where the residual remains within a designated steady-state tolerance ($\delta_{st}=0.03$):

(24) $$\eta =\frac{1}{N}\sum_{i=1}^{N}I\left(\left|e_{x,i}\right|\leq \delta_{st}\wedge \left|e_{y,i}\right|\leq \delta_{st}\right)\times 100\%$$

**Figure 17:**
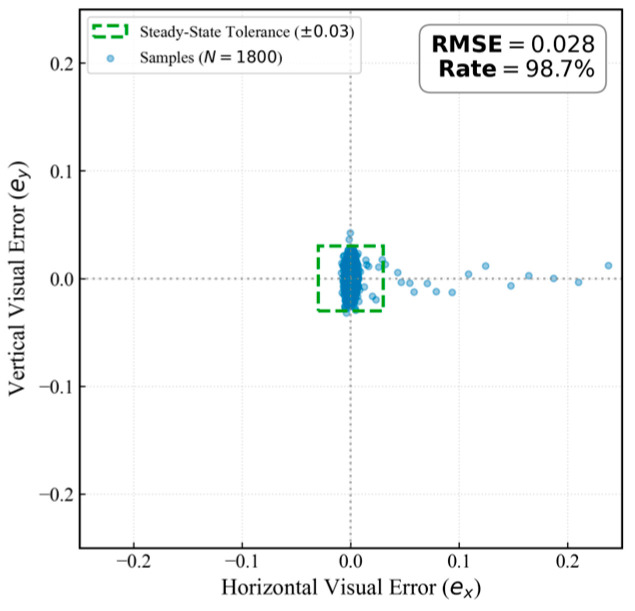
Distribution of steady-state dynamic residuals.

Figure 17 visualizes the error distribution derived from the stable tracking phase (Exp. II in Table 5). As illustrated, the residual sample points are densely clustered around the origin, yielding a normalized dynamic residual RMSE of 0.028. This metric strictly quantifies the active oscillations beyond the intrinsic dead-zone ($\delta_{th}=0.03$), establishing an effective total error envelope of approximately 0.058. Under the proposed velocity mapping mechanism, the horizontal physical tracking speed reaches its operational maximum at a normalized deviation of 0.5. Therefore, when target dynamics remain within the system’s normal operational capacity, the steady-state error is inherently constrained to a minimal numerical scale, restricting the target near the image center. Furthermore, the system achieves a 98.7% Tracking Convergence Rate. These statistics confirm that the ADVS strategy effectively suppresses steady-state chattering and consistently maintains the target within the locking region.

These outcomes are partly attributed to the spatial constraints of the test environment, which necessitated quasi-static phases in the trajectory. During intervals where the UAV remains stationary or the tracking deviation falls within the dead-zone (≤$\delta_{th}$), the dynamic residual is recorded as zero, and the system state is classified as converged.

#### 4.4.3. Vertical Asymmetric Control Analysis

Unlike planar motion, vertical displacement primarily manifests as variations in the bounding box area, which are inherently subtle in 2D video feeds and difficult to quantify via visual observation alone. Therefore, the efficacy of the asymmetric control strategy is validated directly through quantitative Z-axis response data. Adopting the vision-based object following framework established by Pestana et al. [47], this paper utilizes the Normalized Area Error ($e_{\mathrm{area}}$) to evaluate vertical tracking precision independent of absolute depth:

(25) $$e_{\mathrm{area}}\left(t\right)=\frac{S\left(t\right)-S^{*}}{S^{*}}$$

where $S\left(t\right)$ represents the real-time bounding box area (in pixels), and $S^{*}$ denotes the preset reference area (set to 12,000 pixels based on the optimal tracking distance). This dimensionless metric effectively eliminates scale variance arising from initial position offsets, facilitating an objective comparison between different gain strategies.

**Figure 18:**
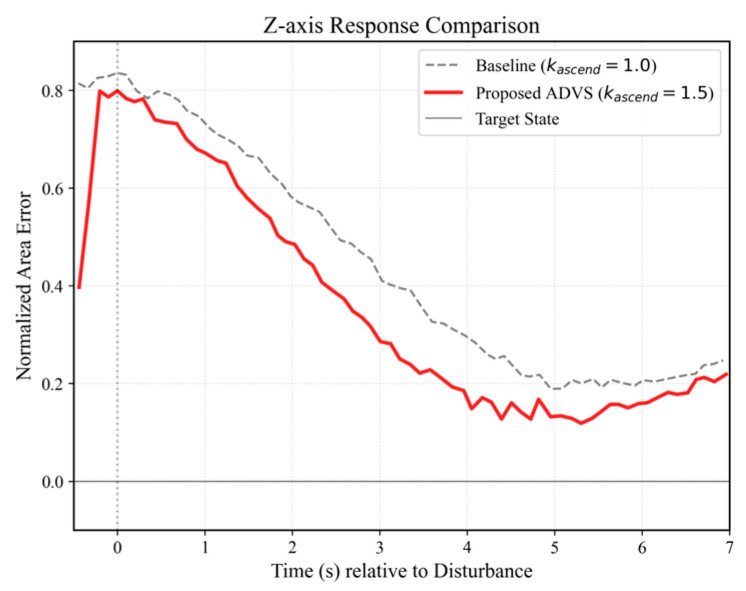
Z-axis Response Comparison. The vertical dotted line at t = 0 marks the onset of the maximum disturbance.

Figure 18 illustrates the comparative step-response trajectories, where the time axis is aligned at t = 0 to mark the onset of a maximum disturbance (initial error $e_{\mathrm{area}}\;\approx \;0.8$, indicating the target is too close). As shown, the Proposed ADVS (red solid line) exhibits a significantly steeper error reduction slope compared to the Symmetric Baseline (gray dashed line). This dynamic behavior confirms that the amplified ascending gain ($k_{\mathrm{ascend}}\;=\;1.5$) successfully compensates for gravitational resistance during the retreat maneuver, providing the necessary thrust to eliminate steady-state errors rapidly. Furthermore, the smooth convergence of the error trajectory validates that the gain configuration provides sufficient damping to prevent actuator saturation while maintaining stability. Despite minor steady-state offsets attributed to environmental perturbations (e.g., incidental target motion), the ADVS strategy consistently demonstrates a faster convergence rate and superior tracking accuracy compared to the baseline method.

### 4.5. System Robustness and Stability Evaluation

#### 4.5.1. System Connection and Initialization Testing

To quantify system robustness under visual occlusion, comparative flight tests were conducted, benchmarking the proposed velocity-based ADVS (integrated with FSM) against a traditional position-based control baseline.

**Figure 19:**
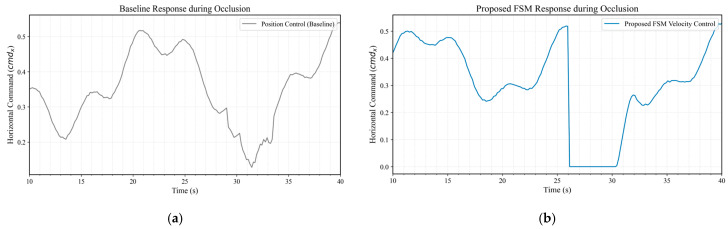
Control response under visual occlusion (occlusion occurring at 25–26 s): (**a**) Baseline system; (**b**) Proposed FSM-based system.

As illustrated in Figure 19, where simulated occlusion was introduced approximately between t = 25 s and t = 26 s in two independent flight sorties, the control behaviors exhibit a critical divergence. In the baseline system (Figure 19a), the control output exhibits a slow decay after the target is lost, failing to decelerate effectively due to the lack of an immediate interrupt mechanism. This residual control signal causes the UAV to continue drifting, increasing the risk of collision. In contrast, the proposed strategy (Figure 19b) leverages the FSM to detect the “Lost” state within a single control cycle and immediately clamps the velocity command to zero $\left(v_{cmd}=0\right).$ Consequently, the control signal drops instantly, eliminating residual inertial commands much faster than the baseline. While the command recovers rapidly after the target is re-locked, the primary advantage lies in this instant cut-off mechanism, which ensures the UAV decelerates rapidly to a safe state upon visual loss.

#### 4.5.2. Steady-State Chattering Suppression

To quantitatively evaluate control output stability, Signal Variance ($\sigma^{2}$) is introduced as the primary metric for characterizing high-frequency actuation chattering. Variance objectively reflects the deviation of velocity commands from the ideal steady state (i.e., zero velocity), calculated as:

(26) $$\sigma^{2}=\frac{1}{N}\sum_{i=1}^{N}{\left(u_{i}-\overset{‾}{u}\right)}^{2}$$

where $u_{i}$ denotes the velocity command (${\mathrm{cmd}}_{x}$ or ${\mathrm{cmd}}_{y}$) at sampling point i, and $\overset{‾}{u}$ represents the mean command value within the observation sequence. This statistical approach effectively decouples stochastic sensor noise from the deterministic system response, providing a mathematical basis for verifying the noise-filtering efficacy of the non-linear dead-zone logic.

**Figure 20:**
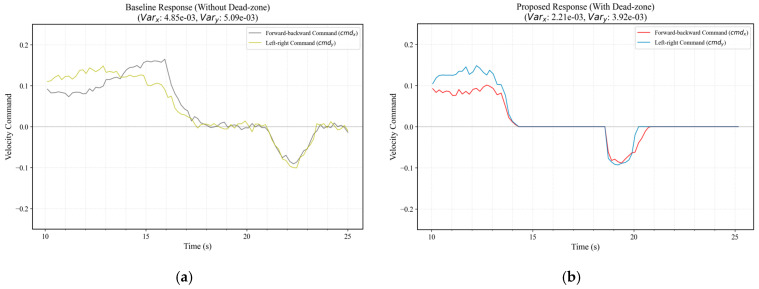
Steady-state chattering suppression analysis: (**a**) Baseline response (without dead-zone); (**b**) Proposed response (with dead-zone logic).

Figure 20 presents the comparative response analysis under identical environmental conditions. As shown in Figure 20a, the baseline system exhibits persistent high-frequency chattering across both longitudinal and lateral channels, with variances reaching $4.85\;\times {\;10}^{-3}$ and $5.09\;\times \;10^{-3}$, respectively, indicating that the actuators are constantly responding to stochastic feedback noise, leading to unnecessary energy consumption and mechanical wear. In contrast, the proposed strategy (Figure 20b) demonstrates distinct zero-command intervals when the tracking error falls within the dead-zone threshold. Quantitative analysis reveals that the command variances for ${\mathrm{cmd}}_{x}$ and ${\mathrm{cmd}}_{y}$ are reduced by 54.43% and 22.98%, respectively. Although a transient command shift occurred due to a wind gust in the latter phase, the substantial reduction in overall variance confirms that the dead-zone strategy significantly enhances control determinism and stability in complex real-world environments [48].

#### 4.5.3. Outlier Filtering and Interference Rejection

**Figure 21:**
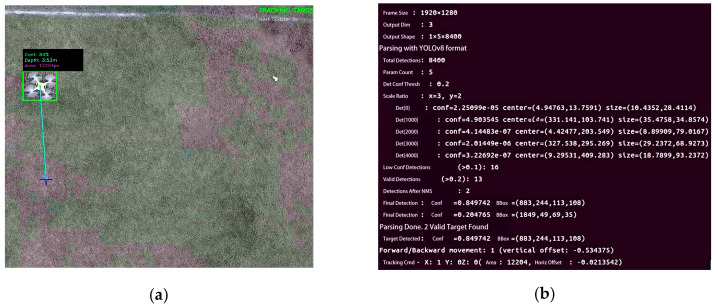
Forward tracking performance: (**a**) Visual recognition interface; (**b**) Edge computing platform terminal output.

To validate system robustness against environmental clutter, a frame-level analysis of the real-time decision-making process was conducted using edge computing logs, as presented in Figure 21b. The log data captures a critical scenario where the visual detection module identified two candidate targets simultaneously—a typical occurrence in lightweight edge detection pipelines where background texture patterns often induce false positives [49].

The system executed the proposed Geometry-Aware Robust Perception Strategy based on Multi-Criteria Fusion. As visible in the right periphery of Figure 21a, an artificial distractor was introduced, which the edge computing log (Figure 21b) captures as a candidate. Rather than relying solely on probabilistic thresholding, the rejection was enforced by the deterministic physical constraints defined in the previous section. Specifically, the bounding box area (S = 2415 px) fell significantly below the minimal threshold defined by the Inverse-Square Geometric Scale Constraint, correctly classifying it as distant background clutter, while the calculated aspect ratio ($r_{i}$ ≈ 1.97) violated the Morphological Shape Constraint, deviating from the typical rigid-body geometry of the target UAV. Consequently, the fusion logic effectively filtered this high-distortion outlier, retaining only the valid target to generate precise motion commands.

### 4.6. Comparison with State-of-the-Art Methods

To objectively evaluate the engineering performance and practical applicability of the proposed system, a comparative analysis was conducted against recent State-of-the-Art (SoA) solutions, as detailed in Table 6.

While heavy-weight Transformer-based architectures (e.g., TDAT, BRA-YOLOv10) offer high tracking robustness, their dependence on desktop-grade GPUs necessitates ground station processing, introducing communication latency incompatible with close-range maneuvering. Conversely, lightweight edge trackers (e.g., T-SiamTPN) often compromise stability due to insufficient refresh rates (7.1 FPS). In contrast, the proposed onboard solution utilizes the Jetson Orin NX to achieve a stable frequency exceeding 30 FPS, satisfying strict real-time control requirements without offloading. Furthermore, addressing the architectural bottleneck of standard ROS-based frameworks—which exhibit latencies around 98.3 ms due to middleware serialization—the proposed “Deep Integration” via DJI PSDK establishes a middleware-free architecture that reduces total latency to approximately 29 ms. This reduction of over 60% enables instantaneous reaction to disturbances. Finally, distinguishing itself from methods relying solely on bounding box accuracy (e.g., ADG-YOLO), the system integrates the ADVS control algorithm with FSM logic to provide control-layer robustness, effectively compensating for the inherent pixel-level jitter of visual sensors.

### 4.7. Summary of Quantitative Results

To rigorously quantify the system’s performance metrics, the validated operational boundaries are summarized in Table 7. These parameters represent the synthesized optimal performance envelope established through the comprehensive field trials described in Section 4.3.2. It is important to clarify that these operational ranges are not derived from a single sortie; rather, they represent the aggregated optimal results verified across multiple experimental sets, confirming the system’s adaptability to diverse flight conditions.

## 5. Discussion

### 5.1. Performance Analysis and Metric Adequacy

The experimental results demonstrate that the proposed system achieves an optimal balance between tracking accuracy and real-time responsiveness. To rigorously validate these engineering improvements, specific metrics were selected to quantify the system’s dynamic behaviors.

1. Vertical Dynamic Response;

The Normalized Visual Error Magnitude ($∥e\left(t\right)∥$) served as the critical benchmark for comparing the proposed ADVS strategy against the standard PID baseline. Rather than merely assessing static accuracy, the analysis of this metric confirms that the ADVS strategy achieves significantly faster convergence rates and rapid stabilization. This validates that the asymmetric gain scheduling effectively enhances system responsiveness, while the integrated dead-zone logic accelerates the settling process.

2. Stability and Precision;

The introduction of Signal Variance ($\sigma^{2}$) and RMSE provides a comprehensive measure of control smoothness and tracking accuracy. The significant reduction in variance confirms that the dead-zone logic effectively filters out high-frequency sensor noise. Simultaneously, the low RMSE validates the ADVS strategy’s capability to consistently maintain the target at the image center during real-time tracking, ensuring robust locking precision.

3. Perception and System Latency;

In terms of perception, the Geometry-Aware Strategy enforces kinematic consistency to purify control inputs against environmental clutter. Furthermore, the middleware-free PSDK architecture minimizes end-to-end latency to approximately 29 ms, providing a critical safety margin for handling sudden disturbances.

### 5.2. Limitations and Future Directions

While the system demonstrates robust performance in typical operational scenarios, three primary limitations are acknowledged. First, regarding the evaluation methodology, the current analysis relies on onboard telemetry and relative visual feedback. Since the control objective of the proposed IBVS framework is to minimize error in the image plane, the evaluation primarily focuses on the relative visual tracking accuracy and dynamic response. While absolute geodetic positioning accuracy (via RTK-GPS) is not the primary metric for visual servoing stability, it is acknowledged that external ground truth could provide supplementary trajectory analysis in future works. Second, regarding system capability, the reliance on a single RGB sensor makes the system vulnerable to extreme lighting conditions (e.g., night, strong backlight), where the target may drift beyond the fixed field of view. Third, the experimental scenario design was constrained by new low-altitude aviation regulations in our region (effective 1 January 2026). The strict geo-fencing constraints necessitated the inclusion of quasi-static phases and limited dynamic velocities. Consequently, these operational restrictions inevitably influenced the statistical results, as the recorded dataset inherently includes a certain proportion of restricted motion phases dictated by safety compliance rather than algorithmic performance alone.

To address these challenges, future research will focus on two strategic directions: integrating Multi-modal Sensor Fusion (e.g., Thermal or LiDAR) to enhance all-weather adaptability, and implementing Trajectory Prediction Networks (e.g., LSTM or Kalman Filter) to estimate target motion during extended occlusions, thereby extending the system’s re-acquisition capability in complex environments. Furthermore, leveraging recent advancements in AI-based control and networking algorithms [52], we aim to explore deep reinforcement learning policies to further optimize tracking robustness against stochastic dynamic disturbances.

## 6. Conclusions

This paper presents the design and implementation of a real-time visual tracking system for UAVs based on PSDK, explicitly addressing the challenges of deploying high-speed visual servoing on resource-constrained edge platforms. Distinct from traditional paradigms that rely heavily on scaling visual model complexity, this study validates a crucial insight: a standard lightweight detector (e.g., YOLOv8), when synergized with a mathematically rigorous control architecture, can achieve high-precision tracking performance.

By establishing a “Deep Integration” architecture via the DJI PSDK, the system eliminates the non-deterministic latency characteristic of middleware layers, securing a deterministic end-to-end response of approximately 30 ms. Crucially, the proposed Lightweight Asymmetric Decoupled Visual Servoing (ADVS) strategy, integrated with Finite State Machine (FSM) logic and adaptive dead-zones, effectively compensates for the stochastic jitter inherent in edge perception, ensuring system stability even during aggressive maneuvers.

Extensive field trials verify the system’s practical deployment capability, maintaining a robust lock on targets at speeds up to 23 m/s and distances extending to 125 m. Ultimately, these results substantiate a distinct conclusion: high-performance aerial tracking on edge devices does not rely solely on the sophistication of detection algorithms. Rather, superior tracking capabilities can be effectively achieved through the architectural minimization of latency and the precise compensation provided by specialized control strategies.

## Figures and Tables

**Table 1:** Comparison of Advanced Detection Architectures for Edge UAV Applications.

Model	Architecture	Edge Inference Latency	Ecosystem Maturity	Small Object Performance	Key Limitation
RT-DETR [18]	Transformer	High ($O\left(N^{2}\right)$)	Low (Memory Heavy)	High	Computational Cost
YOLOv5 [20]	CNN (Legacy)	Low	Very High	Moderate	Feature Aggregation
YOLOv10 [19]	CNN (NMS-free)	Variable (Optimization Gap)	Medium (Experimental)	Moderate	TensorRT Support
YOLOv11 [20,21]	CNN	Medium	Medium	Comparable to v8	Diminishing Returns
YOLOv8 [20,22]	CNN (Selected)	Low	High (Standard)	Robust (C2f)	-

**Table 2:** System Latency Comparison: Traditional ROS vs. Proposed PSDK Integration.

Performance Metric	Traditional ROS Architecture	Proposed Deep Integration
Driver Mechanism	High Latency (Default 16 ms Timer) [23]	Real-time (Direct Access)
Communication Overhead	High (Serialization & Protocol Stack) [24]	Minimal (Raw Binary Transfer)
Total System Latency	>60 ms (Theoretical Estimate)	~30 ms (Experimentally Measured)

**Table 3:** Control Parameters Configured for the Experimental System.

Parameter	Symbol	Value	Rationale for Configuration
Control Loop Frequency	$f_{ctrl}$	25 Hz	Matches vision inference rate.
Confidence Threshold	$c_{th}$	0.7	Balances detection precision and recall.
Dead-Zone Threshold	$\delta_{th}$	0.03	Exceeds noise floor to suppress chattering
Search Yaw Rate	$\omega_{search}$	0.2 rad/s	Optimizes FOV scanning coverage.
Safety Timeout	$T_{timeout}$	2000 ms	Bridges temporary target occlusions.
Stationary Velocity	$v_{static}$	0.1 m/s	Verifies physical stabilization.

**Table 4:** Validation Scope: Target Specifications and Challenging Conditions.

Dimension	Item/Condition	Key Specifications/Description	Selection Rationale & Robustness Mechanism
I. Target Heterogeneity	DJI Mini 2 (Micro)	Weight: <249 g Size: 138 mm Low inertia, wind-sensitive	Sensitivity & Stability Test: 1. Size: Validates detection limits (small pixel occupancy). 2. Dynamics: Tests dead-zone logic against wind-induced high-frequency jitter.
	DJI Phantom 4 (Standard)	Weight: 1380 g Size: 350 mm Balanced inertia and speed	Standard Baseline: Benchmark for calibration and steady-state accuracy validation.
	DJI Inspire 2 (Medium)	Weight: 3440 g Size: 605 mm High inertia, high top speed	Boundary & Response Test: 1. Size: Tests robustness against FOV boundary exits due to large size. 2. Speed: Validates tracking lag minimization during rapid maneuvers.
II. Environmental Complexity	Illumination	Ideal Front-lighting Strong Back-lighting	1. Visual Robustness: Tests tracking stability under glare and silhouette effects.
	Wind Disturbance	Crosswind (~8 m/s)	2. Control Robustness: Validates lateral drift compensation (ADVS) under Level 4–5 winds.
	Background	Complex Clutter	3. Perception Robustness: Tests outlier rejection against texture-rich backgrounds (trees/grass).

**Table 5:** Consolidated Field Experimental Protocols and Evaluation Metrics.

Exp. Category	Experimental Scenario & Operational Protocol	Evaluation Metrics
I. Planar Tracking (Section 4.4.1)	Step-Response Test: Target executes rapid acceleration and braking maneuvers to induce sudden visual deviation.	$\mathrm{Settling}\;\mathrm{Time}\;\;\left(T_{s}\right)$ $\mathrm{Normalized}\;\mathrm{Visual}\;\mathrm{Error}\;\mathrm{Magnitude}\;(∥e\left(t\right)∥)$
II. Steady-State (Section 4.4.2)	Stable Phase Analysis: Continuous tracking analysis over a duration of approx. 60 s (1800 frames) under stable operation conditions.	Root Mean Square Error (RMSE) Dead−zone Rate (η)
III. Vertical Control (Section 4.4.3)	Asymmetric Gain Validation: Target performs rapid ascent (gravity-opposed) and descent (gravity-assisted) maneuvers.	$\mathrm{Normalized}\;\mathrm{Area}\;\mathrm{Error}\;(e_{\mathrm{area}}$)
IV. Robustness (Section 4.5.1, Section 4.5.2, Section 4.5.3)	A. Occlusion: Target intentionally blocked for approx. 3 s (starting at t ≈ 25 s) to test FSM clamping.	Deceleration Response
	B. Chattering: Verification of actuator stability during steady-state hovering.	$\mathrm{Signal}\;\mathrm{Variance}\;(\sigma^{2})$ winds.
	C. Outlier: Injecting false positive targets to test filtering logic.	Classification result

**Table 6:** Comparison of system performance metrics and architectural characteristics with state-of-the-art UAV tracking methods.

Method /System	Core Framework	Hardware Platform	End-to-End Latency	System Frequency	Critical Limitation
BRA-YOLOv10 [5]	YOLOv10 + Attention	PC Workstation (RTX 3090)	N/A	118 FPS	Requires Ground Station; High computational cost.
ADG-YOLO [50]	YOLOv8-Tiny (Pruned)	Edge Device (Lubancat4)	N/A	27 FPS	Inefficient optimization; Lower FPS despite model pruning.
TDAT [4]	Transformer + Global Agent	PC Workstation (RTX 2080Ti)	N/A	34.5 FPS	Requires Ground Station; Heavy attention mechanism load.
ROS 2 System [14]	ROS 2 (DDS) + YOLOv8-Seg	Laptop (Intel i7 CPU)	98.3 ms	28.7 FPS	Requires Ground Station; Middleware serialization latency.
T-SiamTPN [51]	Siamese Transformer	Edge Device (Jetson Nano)	N/A	7.1 FPS	Low FPS; Insufficient for high-speed maneuvering.
Proposed System	YOLOv8 + PSDK (Direct)	Edge Device (Jetson Orin NX)	~29 ms	>30 FPS	Balanced; Optimized for onboard real-time control.

**Table 7:** Validated operational envelope of the proposed tracking system.

Test Parameters	Test Range	Description
Flight Altitude	5–15 m	Covers typical low-altitude operational heights.
Relative Tracking Range	2–15 m	Effective detection distance between tracker and target.
Max Sortie Length	>125 m	Validated continuous tracking distance (limited by operational field dimensions and national regulations).
Target Flight Speed	0–23 m/s	Includes hovering, low-speed and medium-speed maneuvers.
Tracking Distance	5–125 m	Effective operational range.
Target Manoeuvring Modes	Straight, Turning, Hovering	Verifies multi-directional tracking capability.

## Data Availability

The data presented in this study are available on request from the corresponding author. The data are not publicly available due to restrictions within the project’s confidentiality agreement.
